# Data-to-music sonification and user engagement

**DOI:** 10.3389/fdata.2023.1206081

**Published:** 2023-08-10

**Authors:** Jonathan Middleton, Jaakko Hakulinen, Katariina Tiitinen, Juho Hella, Tuuli Keskinen, Pertti Huuskonen, Jeffrey Culver, Juhani Linna, Markku Turunen, Mounia Ziat, Roope Raisamo

**Affiliations:** ^1^Department of Fine and Performing Arts, Eastern Washington University, Cheney, WA, United States; ^2^Tampere Unit for Computer-Human Interaction (TAUCHI), Tampere University, Tampere, Finland; ^3^School of Business, Eastern Washington University, Spokane, WA, United States; ^4^Department of Information Design and Corporate Communication, Bentley University, Waltham, MA, United States

**Keywords:** data-to-music, sonification, user engagement, auditory display, algorithms

## Abstract

The process of transforming data into sounds for auditory display provides unique user experiences and new perspectives for analyzing and interpreting data. A research study for data transformation to sounds based on musical elements, called data-to-music sonification, reveals how musical characteristics can serve analytical purposes with enhanced user engagement. An existing user engagement scale has been applied to measure engagement levels in three conditions within melodic, rhythmic, and chordal contexts. This article reports findings from a user engagement study with musical traits and states the benefits and challenges of using musical characteristics in sonifications. The results can guide the design of future sonifications of multivariable data.

## 1. Introduction

Music can be a highly engaging art form in terms of pure listening entertainment and, as such, a powerful complement to theater, film, video games, sports, ballet, ceremonies, and sacred rituals. So it seems reasonable to assume music has the ability to capture the focus of people who also listen to data in the form of auditory display. In this article, we will refer to auditory display as *sonification*, “the transformation of data relations into perceived relations in an acoustic signal for the purposes of facilitating communication or interpretation” (Neuhoff, 2011). The following is a research study conducted to establish how musical characteristics contribute to engagement with data-to-music sonification. Our study stems from a 3-year investigation of data-to-music for five companies in Finland that were seeking innovative ways to present data for their employees and customers. The companies were represented by industries related to power generation, smart electronics for medical devices and building monitoring, smart watches, construction, and architecture. The project began with the development of new sonification software for data transformation called D2M. The D2M software was used to create sonifications for the user engagement study to determine the reliability of musical characteristics for enhanced engagement. Engagement offers a key advantage to auditory displays of data if the roles of musical characteristics and engagement are understood correctly.

## 2. Background

### 2.1. Sonification

The use of musical elements in sonification has been formally explored by members of the International Community for Auditory Display since the mid-1990s (Kramer et al., 1999; Hermann et al., 2011). The interest in incorporating musical characteristics in sonification can be summarized from the study by Brown et al. (2003): “the use of musical sounds has been recommended because of the ease with which musical sounds are perceived.” A summary of music-related research and software development in sonification can be obtained from the study by Bearman and Brown (2012). Earlier attempts were made by Pollack and Ficks (1954), yet according to Vickers (2017), and other researchers, such as Walker and Nees (2011), “questions of aesthetics and musicality remain open in the field of sonification,” and therefore the path for music in sonification remains uncertain. This impression shows how challenging an interdisciplinary area of research can be. As an example, Vickers, Hogg, and Worrall submit that the dual nature of music and analysis is hard to achieve: “A major design challenge is to create sonifications that are not only effective at communicating information but which are sufficiently engaging to engender sustained attention” (Vickers et al., 2017). It appears from this statement that the process of interpreting data with musical qualities can contribute to enhanced levels of engagement but also distract from the analysis. As an example, the concern can be seen in a condition monitoring study by Hildebrandt et al. (2016), which did not apply musical contexts due to concerns of continuous monitoring fatigue with music. Their study reveals how researchers perceive risks in using musical sounds, but the authors acknowledge there is a lack of attention to musical aesthetics and “potentially more pleasing designs” for long-term usability and effectiveness. Meanwhile, Vickers (2017) suggests the path toward successful sonifications with musical elements can be assisted by the knowledge and experience of composers by recommending “an aesthetic perspective space in which practice in various schools of music composition might be used to improve the aesthetic design and interest of sonifications.”

It is important to note that the use of musical characteristics in sonification can serve multiple purposes. To start, musical expressions that relate to aesthetics can enhance the user experience, and this can strengthen the perceptual experience with data. Positive user experiences can translate to more time spent with data analysis and improve interpretations. Finally, the mapping possibilities to musical traits are numerous, and this brings opportunities to associate multivariate data with different types of musical features, forms, and expressions.

The study reported in this article was carried out in a project called Data-to-Music, which focused on the development of custom-made software to map multivariate data with musical characteristics. The data came mostly from monitoring the conditions of buildings, machines, weather, fitness tracking, and athletic experiences. What made the project unique was the focus on the user experience with music. The project sought to design the most effective sonifications with only musical elements rather than any sounds (synthetic, nature, auditory icons, or earcons) (Brewster, 2009).

In this article, we first outline our sonification approach with musical characteristics and engagement as priorities, and then describe the surveys we used to evaluate user engagement. The auditory features for the surveys relied on D2M software to transform weather data into musical elements and expressions in melodic, chordal, and rhythmic contexts.

### 2.2. Form and function

In this article, we are guided by the research question “Can musical characteristics contribute to meaningful data perception, analysis, and interpretation?” The challenge is to make sure the aesthetic qualities of musical sounds will maintain or contribute to the integrity of coherent information in the auditory display. We submit that data-to-music sonification can not only engage users but also maintain coherence as a foundation for understanding combinations of musical characteristics.

In addressing this challenge, we venture into the human–computer interaction (HCI) domain of form and function or aesthetics and usability (Norman, 2004; Tractinsky, 2006). Various studies have revealed that this duality is most likely interconnected to the extent that a positive perception of usability enhances our perception of aesthetics, and aesthetic appeal can enhance our perception of usability (Tractinsky et al., 2000; Tuch et al., 2012). This association suggests an aesthetic appeal from musical traits could contribute to increased usability and therefore indicates some potential advantages for enhancing the quality and quantity of time users could dedicate to experiences and interactions in data-to-music. The interpreting experiences with the aid of musical characteristics offer new perspectives for the exploration of data through a relatively new form of mediation (with musical ideas) between humans and data (Dillon, 2006). From this interaction within arcs of exploration and interpretation, one would hope for a meaningful connection *via* engagement to help users analyze information from data. Engagement's significance for analysis and its inherent connections to usability and aesthetics will be addressed later in the paper.

## 3. D2M software for data-to-music sonification

To test the possibilities of musical characteristics for data representation and display, our research team designed, and programmed the D2M data-to-music mapping software. The software enables the users to map variables from time series datasets to musical characteristics in independent tracks we call “streams.” In each track, users can make selections for instrument type (or timbre), pitch range, rhythmic complexity, accent chords, articulation, and loudness. The user can also modify the duration of the generated sonification, and the data can be scaled accordingly. The wide selection of mapping options provides users with opportunities to hear data from many musical attributes. The musical structures are generated as MIDI output from the data content in combination with mapping selections for each stream. Streams can represent one or many datasets from a data bank, e.g., one stream might represent cloud cover and another stream might represent wind speed, and users could hear these combined.

Additional tools include the possibility of setting thresholds to filter out segments of the data. There are also preprocessing tools for linear and logarithmic scaling and a tool to invert data values. The high degree of flexibility of the tools enabled the research team to create auditory displays that sound composed, even though the results were determined by the data and algorithms.

While the algorithms for the D2M software were researched and developed over 3 years at TAUCHI (Middleton et al., 2018), the designs were informed by 10 years of experiences from users and composers with the *music algorithms* Web-based software (https://musicalgorithms.org/4.1/app/#) (Middleton and Dowd, 2008; Bywater and Middleton, 2016). The data for testing and developing the D2M software were provided by companies with the goal of developing proof-of-concepts that would allow users to hear and interpret data through musical forms of expression. The D2M development project was funded by a Tekes innovation grant.

## 4. User engagement

Multiple studies have shown that the task of defining and measuring engagement is quite complex, and many evaluations are connected to the field of education to understand how students are engaged in learning (Lutz Klauda and Guthrie, 2015). In various studies on engagement, common descriptive terms, such as motivation, persistence, and effort, emerge. Lutz Klauda and Guthrie (2015) elegantly separate motivation as goal-oriented, based on values and beliefs, a mode of “behavioral displays of effort, time, and persistence in attaining desired outcomes.” Additional attributes from other studies include focused attention, curiosity, novelty, and challenge (Webster and Ahuja, 2006; O'Brien and Toms, 2008).

The most seminal work on engagement evaluation appears to be by O'Brien and Toms (2008, 2010). The O'Brien and Toms study defines engagement in extensive detail by building on the premise that engagement is a process in three stages, namely, point of engagement, sustained engagement, and disengagement, and as the process unfolds in time, there are multiple layers of experience called threads. The categorization of threads is derived from the work of McCarthy and Wright (2004).

The O'Brien and Toms study from 2008 sought to refine the threads of experience: Compositional (narrative), spatiotemporal, emotional, and sensual, into six key factors for engagement. Their research generated questionnaire items for a User Engagement Scale (UES) consisting of 31 statements associated with the six engagement factors (O'Brien and Toms, 2010). O'Brien and Toms (2008, 2010) built their engagement model mainly from visual displays related to Web searching, video games, online learning, and online shopping. Our Data-to-Music study adopted the O'Brien and Toms' engagement instrument from 2008 to 2010 to measure user engagement from musical characteristics and contexts in auditory displays of data. In O'Brien et al. (2018) published an updated user engagement scale called a “short-form framework” with an attempt to consolidate the six engagement factors down to four factors. They also sought to reduce the number of questionnaire statements from 31 to 12. Our user engagement study for data-to-music was already in progress by this time, so we will report findings based on the long-form UES with six factors (*focused attention, perceived usability, aesthetics, endurability, novelty*, and *felt involvement*) and 31 statements. In addition to demonstrating engagement levels from musical characteristics in sonifications, we hope our study, based on the UES long form, will provide some observations and comparisons to inform the UES short form's objectives. In particular, our results may provide insights toward the decision to consolidate UES factors for *endurability, novelty*, and *felt involvement* into one factor called “reward” (O'Brien et al., 2018).

## 5. Methods

### 5.1. Three engagement surveys

From 2017 to 2018, three user engagement surveys were conducted with 72 human subjects. The purpose of the surveys was to capture the impact of basic musical characteristics, such as pitch, rhythm, and timbre, in auditory displays of data in the contexts of melodic, chordal, and rhythmic sonifications.

There were 24 participants per study (*N* = 72). Participants' ages ranged from 18 to 56 years. Nearly 53% had more than a year of musical training, but only 29% of all participants considered themselves semi-professional or professionally trained musicians. Nearly 78% of participants across the three studies were unfamiliar with data-to-music sonification based on the question “Have you heard data sonifications before this experiment.” Additional background information includes 33 male participants and 38 female participants, and one was of a non-specified gender. All participants rated how they were feeling as they started the surveys based on five descriptions, namely, sad, a bit sad, neutral, a bit happy, and happy. All were within the range of neutral to happy.

### 5.2. Three musical contexts

Three surveys were designed to place participants in the context of either melody, chords, or rhythm as defining features for data mapping. In converting data to melodic contexts, the focus was on pitch mapping, where high and low numeric data values were mapped to high and low pitches. In this context, the auditory display unfolds in a linear, melodic line of sound. In mapping to the context of chords, pitch mapping is executed in the same manner as melody, but an array of tones is added vertically to the melodic line. In D2M, chords are featured as a composite sound of two to four pitches perceived as one sonority (or sound entity), and these vertical sonorities flow in a harmonic sequence. The Chord-based survey used standard usage of dyads, tetrachords, and triadic combinations of tones that are found in tonal Western music, but the study did not attempt to apply harmonic principles that relate to harmonic syntax, semantics, or voice leading (Aldwell and Schachter, 2011). Instead, the D2M algorithm mapped data to generic chord structures with some variability of chord tone density and attack, i.e., the chords could be presented as a simultaneity with two, three, or four tones all at once or rolled with a quick linear unfolding of the chord.

The context of Rhythm featured rhythmic mapping, i.e., data mapped to rhythmic durations assigned to a uniform noise or pitched sound. A data point could be mapped to a rhythmic value or a rhythmic motive. In general, a set of low data points would generate long rhythmic durations (showing slow activity), and by contrast, a set of high data points would generate short rhythmic durations (showing accelerated activity).

Although rhythm was a featured context for one survey, it was also one of three conditions for all three surveys. The musical context of Rhythm and the use of rhythm as a key element for a survey condition were not mutually exclusive, so in order to provide some differentiation, the Melody and Chords surveys used a moderate scope of rhythmic activity as a condition, and a more wide scope of rhythmic activity was used for the entire Rhythm survey. Details are provided in the conditions section.

### 5.3. Weather data sources and listening tasks

**Figure 1:**
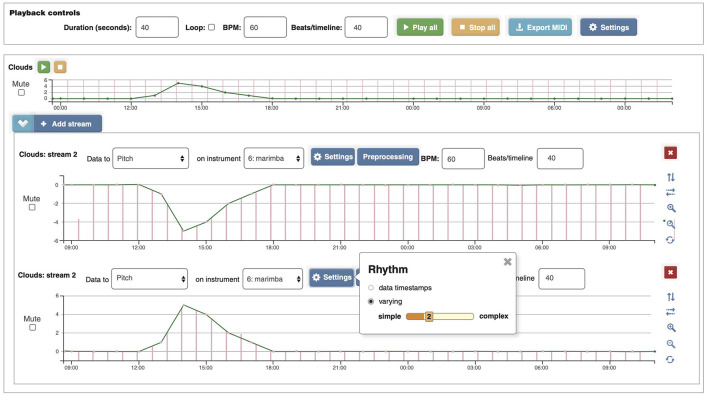
D2M interface showing how clouds data in okta were inverted so that cloud cover would represent low pitch ranges to express “deep” or “heavy” impressions and sunshine would represent high pitch ranges for brightness. The top section represents the data, the middle section represents the inverted data with auditory stream mappings, and the bottom section shows how the auditory stream could have been mapped without the inversion (along with a box showing the rhythmic selection of the moderate level “2” distribution of rhythms). Each result was 40 s in duration with an underlying tempo of 60 beats per minute.

**Figure A1:**
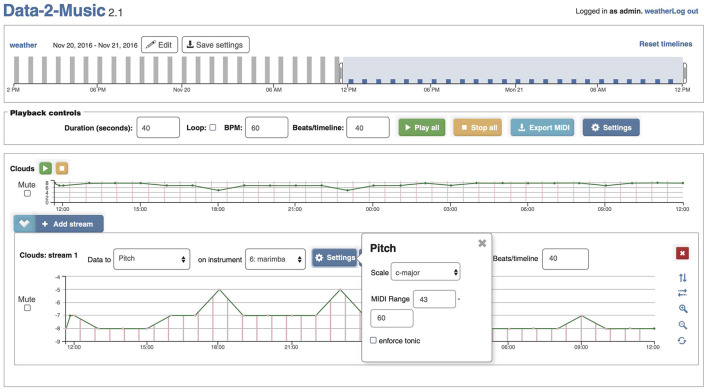
D2M software interface mapping November cloud cover data in oktas to a MIDI range (43–60), instrument (marimba), and musical scale (C major).

The sonifications for the user engagement surveys used data derived from weather forecasting. In the Melody and Chords surveys, the data were derived from cloud cover (called oktas), while the Rhythm survey used data from wind speeds. The wind speed and oktas data came from 24-h periods in three different months of the year, namely, April, May, and November. The data came from the Finnish national weather service database (https://en.ilmatieteenlaitos.fi/statistics-from-1961-onwards). The data from three separate months showed rather diverse weather patterns, and this enabled the studies to present mostly active, moderately active, and mostly static sonification experiences for each condition. As an example, the data for May was more static than that for November (compare Figure 1 with **Figure A1**).

To determine user engagement, the surveys relied on user tasks and statement responses upon completion of tasks. One of the tasks we created was a simple listen and click interface with a question referencing whether you hear clouds or sunshine. We were able to use the D2M data inversion tool to generate pitched results from the data that express amounts of sunshine or cloud cover from the same data source (Figure 1), a technique similar to one described by Walker and Kramer (2005). High okta values for extreme cloud cover would sound low, while low okta values representing sunshine would sound high and bright after the inversion tool was applied. For mapping details, see Appendix.

Wind speeds were used for the Rhythm survey since the mapping process and correlations between wind speeds and rhythm seemed more appropriate than clouds and rhythm.

### 5.4. Three conditions per survey

Each survey featured sonifications under three conditions, which were differentiated by the order of complexity among three fundamental musical characteristics. The simplest condition featured plain sounds; the next level of complexity featured tones with timbre; and the most complex condition featured tones with timbre and rhythm.

Three conditions in detail:

(1) Plain sounds, as defined by tones represented by sine waves or a singular semi-pitched percussive noise (from claves: a monotone, partially pitched sound with a woody attack noise). Plain sounds may be referenced as “noise” or “pure tones.” In the Melody and Chords surveys, the plain sounds were guided by one steady rhythmic value of quarter notes (or crotchets) at a moderate tempo. The D2M software refers to this rhythm setting as “level 1.” In the Rhythm survey, percussive noise was used in lieu of pure tones, and the condition featured a “level 4” rhythmic setting (widening the scope of rhythmic options).
(2) Tones with a uniform sound color, called timbre. In this condition, listeners hear the same pitch mapping results as in the plain sound condition, but in this case, there is a timbral color from a marimba added. This condition with a marimba timbre was present in all three surveys. The Melody and Chords surveys maintained the same level 1 rhythmic setting, so that the sounds were guided by a uniform rhythmic value of quarter notes at a moderate tempo. In the Rhythm survey, with a focus on data mapped to rhythms, the condition used the level 4 setting. The pitches for this condition in the Rhythm survey were set to a monotone (singular pitch)—not informed by the data.
(3) Tones with sound color and rhythm based on an elevated rhythmic activity relative to the other conditions. Activity level 2 was used in the Melody and Chords surveys, and level 4 was used for the Rhythm survey. The elevated rhythmic levels increasingly expand the rhythmic results, such as quarter notes, eighth notes, eighth note triplets, and sixteenth notes in a moderate tempo. Although the conditions for the Rhythm survey were quite similar to the Melody and Chords surveys, the third condition varied the most in how the number of rhythmic options at level 4 (instead of level 2) allowed the data to generate a wider array of complex rhythmic results than the other studies. This expansion of options allowed the survey to adhere to an enhanced rhythmic context, e.g., high wind speeds generated very active rhythmic experiences, while slow wind speeds generated very passive rhythmic experiences. The conditions for the Rhythm survey used traditional drum set sounds consisting of cymbal noises and drum tones, representing a degree of timbral variety in the context of rhythmic variety. One can listen and compare all data-to-music audio files for May datasets located in Supplementary material.

### 5.5. Survey tasks and statements

**Figure 2:**
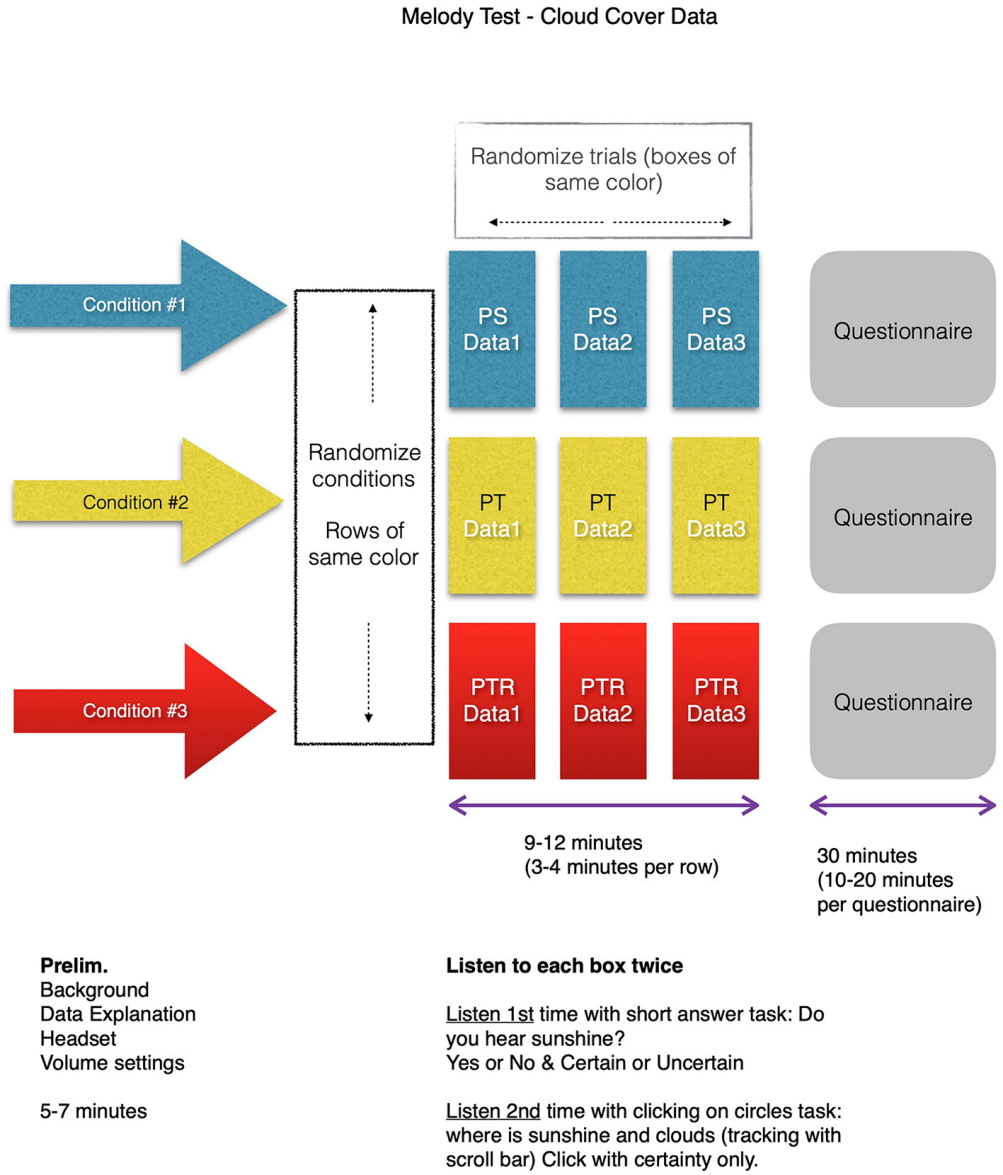
The flow of the Melody survey with cloud cover data from three separate months shown as Data 1, Data 2, and Data 3. Participants listened to each condition with two different tasks (answering a question after listening and then clicking while listening in real-time). Then participants responded to the 31 statements in the UES questionnaire. All three surveys followed the same structure. This flow was repeated three times for each condition. The survey was randomized on two levels, namely, (1) the order of three conditions and (2) the order of three data sonifications within a condition. The conditions were expressed as PS, PT, and PTR, referencing pitched sine waves, pitched timbre, and pitched timbre with rhythm. The randomizations followed an ABC_CBA scheme. The survey duration was about 45min.

The study was designed with two primary tasks, namely, (1) listening tasks with immediate questions (to give users time to experience the sounds and respond in a user-friendly environment) and (2) responding to questionnaire items represented by 31 survey statements (adopted from the O'Brien and Toms, 2008). The surveys' structure and the UES statements are given in Figure 2 and Table 1, respectively.

**Table 1:** Thirty-one statements for the data-to-music user engagement scale (UES).

**#**	**Statement**
1	I was so involved in my listening task that I lost track of time.
2	The time I spent listening just slipped away.
3	My sound experience was rewarding.
4	I felt interested in my listening task.
5	During this sound experience, I let myself go.
6	When I was listening, I lost track of the world around me.
7	These sounds appealed to my auditory senses.
8	I could not identify some of the things I needed to identify from these sounds.^*^
9	I liked the beats and rhythms used in these sounds.
10	If made available, I would continue to listen to these kinds of sounds out of curiosity.
11	I felt frustrated while listening to these sounds.^*^
12	The content of the sounds incited my curiosity.
13	I felt involved in this listening task.
14	Listening to these sounds was worthwhile.
15	I felt in control of my sound experience.
16	I found these sounds confusing to understand.^*^
17	This sound experience was fun.
18	I consider my sound experience a success.
19	These sounds were aesthetically appealing.
20	This sound experience did not work out the way I had expected.^*^
21	The sound layout of these sounds was auditorily pleasing.
22	I felt annoyed while listening to these sounds.^*^
23	I was absorbed in my listening task.
24	These sounds were attractive.
25	I lost myself in this sound experience.
26	I blocked out things around me when I was listening to the sound data.
27	This sound experience was demanding.^*^
28	I felt discouraged while listening to these sounds.^*^
29	I would recommend listening to these kinds of sounds to my friends and family.
30	I was really drawn into my listening task.
31	Understanding these sounds was mentally taxing.^*^

The UES statements are modified from the O'Brien and Toms study from 2010 (Figure A1) to address the context of auditory display. The item number indicates the order in which the statements were presented to the participants. The asterisks indicate that the item was reverse-coded in the same manner as the O'Brien and Toms (2010).

**Figure 3:**
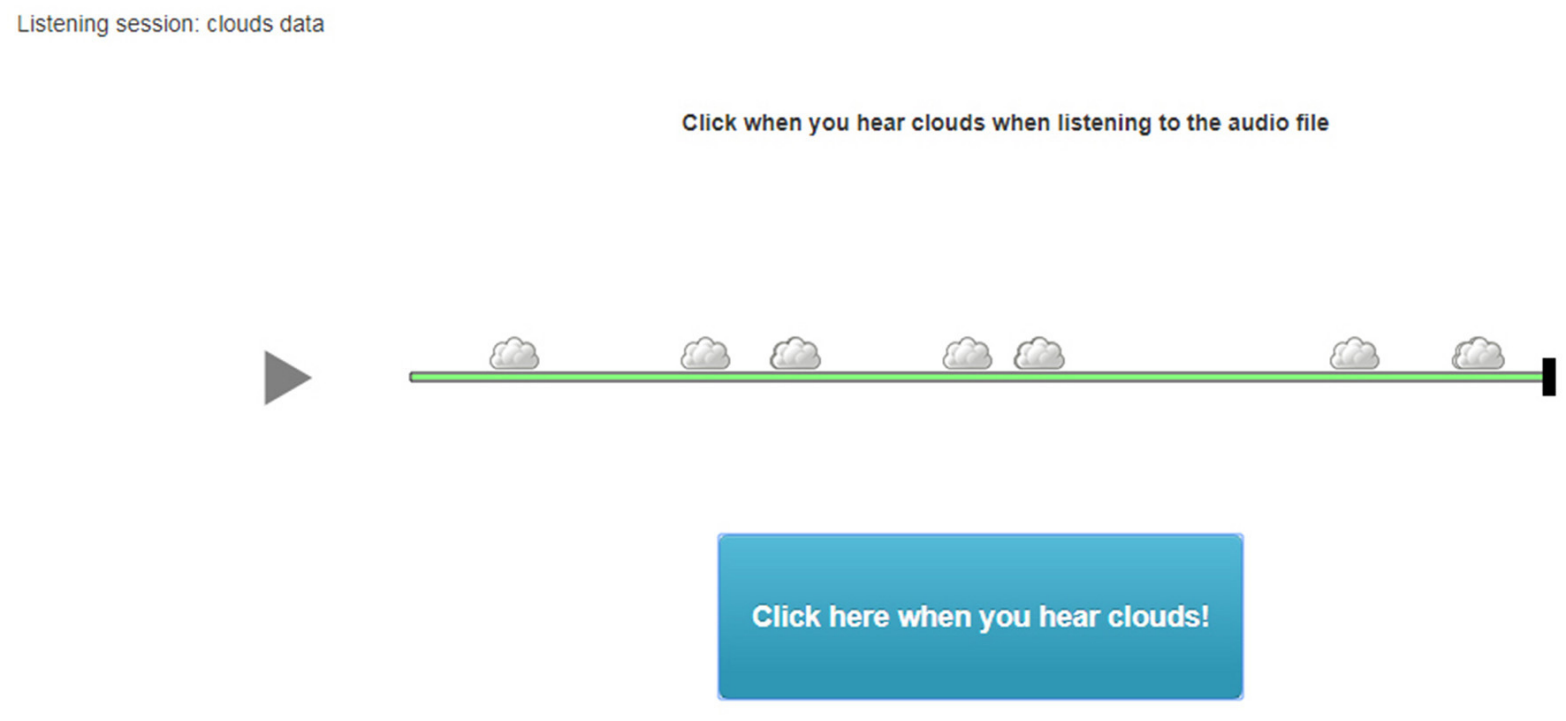
Survey engagement activity. Participants were asked to listen to a sonification, mapped from oktas data, and click when they heard clouds. Upon each click, a cloud would appear at the time point relative to the audio file. The activities of listening and responding to sonifications served as references for measuring engagement, which was the primary focus of the survey. The accuracy of the participants' responses was not measured.

Users responded to the 31 statements after a series of listening tasks related to each condition, namely, (a) plain sounds, (b) tones with sound color, and (c) tones with sound color and rhythm presented *via* ABC-CBA counterbalanced scheme. The 31 statements were completed three times, following the listening tasks with three sonifications (created from data from three different months). The engagement involved listening and clicking when impressions of weather were perceived, for sun, clouds, and wind (See example in Figure 3). The 31 statements cover six different engagement factors called focused attention, perceived usability, aesthetics, endurability, novelty, and felt involvement (O'Brien and Toms, 2010). Responses were measured on a 5-step Likert scale from “strongly disagree” to “strongly agree,” generating data that are ordinally scaled.

### 5.6. Statistical methods for analysis

Survey responses from three conditions, namely, (1) plain sounds, (2) tones with timbre, and (3) tones with timbre with rhythm) were collected from a non-parametric (distribution-free) testing method, and results were calculated by Kendall's Concordance Coefficient—Kendall's W for approximate mean affect, or agreement among raters (to determine low statistical dependency), and Friedman's test for statistically significant differences among median ranked values in paired groups, covering all combinations of three conditions per survey. There were 279 median responses drawn from 31 statements from three conditions across three surveys. Pairwise comparisons were made from the median-ranked results from three conditions for each survey. Friedman's tests were run to compare whether the ordinally scaled data would show differences between the conditions. Statistical significance was determined by the *P*-value being less than the alpha level of 0.05 (*p* < 0.05), which would reject the null hypothesis of no significant difference. Adjusted and non-adjusted significance levels for pairwise comparisons of conditions were explored (the adjusted significance level applies a *Bonferroni* correction to account for the increased likelihood of a rare event when testing multiple hypotheses). We cross-referenced Kendall's W results with Friedman's tests for all three studies to report the most favorable results by order of engagement factors.

## 6. Results

### 6.1. UES results based on fair agreement and statistically significant differences

Friedman's statistical analysis showing adjusted significant differences (*p* < 0.05) combined with Kendall's W tests in the general range of “fair” agreement (0.199–0.383) reveal 14 results across four different engagement factors, namely, focused attention, perceived usability, aesthetics, and novelty (see Table 2). The results below are presented by order of UES engagement factors and statement number(s) from Table 1.

**Table 2:** Results from the data-to-music user engagement scale (UES) surveys based on statistically significant differences from pairwise comparisons from the Friedman hypothesis test (*P* < 0.05).

**Factor of engagement**	**Survey**	**Condition**	**Statement**	**Median value 1–5**	**Average value 1–5**	**Adjusted significant difference**	***P* value (*P < * 0.05)**	**Kendall's W**
Focused attention	Rhythm	Plain sounds	23	4.0	3.75	0.015	0.002	0.260
Perceived usability	Melody	Pitches with timbre and rhythm	15	4.0	3.75	0.012	0.001	0.274
Aesthetics	Melody	Pitches with timbre and rhythm	7	4.0	3.79	0.006	0.000	0.335
Aesthetics	Melody	Pitches with timbre	7	4.0	3.71	0.035	0.000	0.335
Aesthetics	Melody	Pitches with timbre and rhythm	9	4.0	3.83	0.042	0.006	0.215
Aesthetics	Melody	Pitches with timbre and rhythm	19	4.0	3.79	0.006	0.000	0.366
Aesthetics	Melody	Pitches with timbre	19	4.0	3.67	0.018	0.000	0.366
Aesthetics	Melody	Pitches with timbre and rhythm	21	4.0	4.04	0.002	0.000	0.383
Aesthetics	Chords	Pitches with timbre and rhythm	21	4.0	3.83	0.052	0.002	0.270
Aesthetics	Chords	Pitches with timbre	21	4.0	3.75	0.052	0.002	0.270
Aesthetics	Melody	Pitches with timbre and rhythm	24	4.0	3.75	0.015	0.000	0.357
Aesthetics	Melody	Pitches with timbre	24	4.0	3.54	0.007	0.000	0.357
Novelty	Melody	Pitches wit timbre and rhythm	4	4.0	4.33	0.042	0.003	0.249
Novelty	Melody	Pitches with timbre	10	3.5	3.17	0.018	0.002	0.258

#### 6.1.1. Engagement factor for focused attention

There is only one result to report for *focused attention*, and it relates to the Rhythm survey results from pairwise comparisons for statement 23, which showed that listeners found they were more absorbed in their listening tasks when presented with the simplified condition of a plain sound as opposed to the complexities of pitched timbre or pitched timbre with noise.

#### 6.1.2. Engagement factor for perceived usability

There is only one result to report for *perceived usability*, and it relates to the Melody survey results from pairwise comparisons for statement 15, which showed that listeners found they were more in control of the sound experience when presented with pitches with timbre and rhythms as opposed to plain sounds (pure tones).

#### 6.1.3. Engagement factor for aesthetics

There are 10 results to report for *aesthetics*:

□ The Melody survey results from pairwise comparisons for statement 7 showed that listeners found the sounds were more pleasing to their auditory senses when presented with (a) pitches with timbre and rhythm as opposed to plain sounds (pure tones); and (b) pitches with timbre as opposed to plain sounds (pure tones).
□ The Melody survey results from pairwise comparisons for statement 9 showed that listeners found they liked the beats and rhythms of the sounds most when presented with pitches with timbre and rhythms as opposed to plain sounds (pure tones).
□ The Melody survey results from pairwise comparisons for statement 19 showed that listeners found the sounds aesthetically appealing to their auditory senses when presented with a) pitches with timbre and rhythms as opposed to plain sounds (pure tones); and b) pitches with timbre as opposed to plain sounds (pure tones).
□ The Melody survey results from pairwise comparisons for statement 21 showed that listeners found the sound layout to be most pleasing when presented with pitches with timbre and rhythms as opposed to plain sounds (pure tones).
□ The Chords survey results from pairwise comparisons for statement 21 showed two pairs of *marginally* adjusted significant differences of 0.052 and 0.052. Listeners found the layout of the sounds to be auditory pleasing when presented with (a) pitches with timbre as opposed to plain sounds (pure tones) and (b) pitches with timbre and rhythms as opposed to plain sounds (pure tones).
□ The Melody survey results from pairwise comparisons for statement 24 showed that (a) listeners found the sounds more attractive when presented with pitches with timbre as opposed to plain sounds (pure tones); and (b) listeners found the sounds more attractive when presented with pitches with timbre and rhythm as opposed to plain sounds (pure tones).

#### 6.1.4. Engagement factor for novelty

There are only two results to report for *novelty*, and they relate to the Melody survey results from pairwise comparisons for statements 4 and 10. Statement 4 showed that listeners found they were more interested in their listening task when presented with pitches with timbre and rhythms as opposed to plain sounds (pure tones). Statement 10 showed that listeners found they would continue listening to the sounds out of curiosity when presented with pitches with timbre as opposed to plain sounds (pure tones).

## 7. Discussion

### 7.1. Factors and elevated engagement levels

The broadest engagement impact, with musical characteristics, appears to reside in the *aesthetics* factor with the conditions for pitches with timbre and rhythm in the context of melody. Among the statistically significant differences, the results show the median was consistently 4.0 (the average range for engagement from the same data for *aesthetics* was 3.54 to 4.04 on a scale of 1 to 5). The results represent elevated engagement levels in relation to data-to-music, with musical results based on simple, pure tones. Aesthetics is clearly a primary factor in engagement in musical contexts.

The two results to report from the *novelty* factor for engagement had median values of 4.0 and 3.5, or an average engagement result of 4.33 for pitches with timbre and rhythm from statement 4 and an average result of 3.17 for pitches with timbre from statement 10 (in this Discussion, please refer to Table 1 for the wording of all statements). While 4.33 is one of the highest average engagement levels to report among 14 results with statistically significant differences, the average of 3.17 is one of the lowest engagement results, barely over the mean threshold of 3.

The remaining results come from two factors, *perceived usability* and *focused attention*. Statement 15 for *perceived usability* in the melody study had a median engagement result of 4.0 for pitched timbre and rhythm. Statement 23 for *focused attention* in the Rhythm study also had a median engagement result of 4.0 for plain sound (noise). This result is the only one reported from the Rhythm study, and it is a unique case where a plain noise sound was more effective in boosting engagement than a pitched timbre. We attribute this result to the challenges users had with hearing wind data among more complex sounds. A simple sound prevailed over the rich musical environment.

When looking more closely at median and average results, what was most interesting was how timbre seemed to make a significant contribution to engagement, while rhythm only seemed to mildly boost the results. As an example, in Statement 7 of the Melody survey, the average engagement level was 3.71 for pitches with timbre, whereas the average result was 3.79 with rhythm added. We see a similar pattern for statement 19 from the Melody survey, where the average for pitches with timbre was 3.67, and with rhythm added, the average was 3.79. The significance of timbre's contribution to engagement can also be observed from the comparison of results between plain tones and tones with timbre. As an example, in statement 7 of the Melody study, the plain tone average result was 2.71, while the pitch with timbre average result was 3.71. A similar comparison can be made between statement 19, where the plain tone average result was 2.63, and the pitch with timbre result was 3.67. In these cases, timbre, as a musical characteristic, added a full-point enhancement in relation to plain tones. The perceived sound colors provided by timbre (McAdams, 2013) provide enhanced experiences for listeners in the surveys.

In general, the Chords survey had many similar results to the Melody study, with elevated results for pitches with timbre and rhythm; however, after running a stringent statistical analysis for significant differences, we can only report two median results of 4.0 for survey statement 21, with average engagement levels of 3.83 for pitches with timbre and rhythm and 3.75 for pitches with timbre (both related to the aesthetics factor). The results from the Chords survey are based on marginally adjusted significant differences of 0.052 in relation to plain sounds. The Chords study used the same data as the Melody study, so we could anticipate how the results might be similar with more parity among the number of results with statistically significant differences. This did not happen, so we note that the chordal context for the sonifications contributed to results that were too uniform. We would need more research to see why significant differences were harder to achieve than in the melody study.

### 7.2. Global view and further studies

From a global view, one might have expected a full complement of musical characteristics (pitch with timbre and rhythm) to consistently contribute to elevated engagement levels. Instead, the results seem mixed. Timbre seems to contribute more than we expected because the separation in the study's results between pure tones and tones with timbre was noticeable, and the integrity of the assessment was affirmed by pairwise comparisons based on Friedman's analysis (significant differences were observed by the separation between plain sounds and those with timbre).

The Rhythm study had remarkably fewer engagement factors represented, with only three statements from *focused attention* and *perceived usability* showing results with significant differences and only one statement, number 23, meeting our stringent criteria. There was something about the complexities of rhythm and data representation in this study that may have brought the median and average responses to their lowest levels across the three studies. The result from statement 23 in the Rhythm study is intriguing in how it may say something about *focused attention*. In this unique case, a singular noise-based sound with a simple array of varying rhythms, determined by the data, showed the potential to capture our listeners' attention equal to or better than more complex musical events. One of the challenges in the Rhythm study also relates to the complexity of drum set sounds (a combination of noise, timbre, and rhythm) used as one of the conditions—requiring a more holistic listening experience than many participants were able to grasp. This musically complex condition received some of the lowest engagement responses across the three studies, mostly for *focused attention, perceived usability, endurability*, and *felt involvement*. We suspect the participants may have been confused by the complexity of details in a saturated sound environment, making it challenging to interpret the data globally from the speed of rhythmic activity in relation to the speed of wind. One might have anticipated higher levels of engagement from a drum set, as one might perceive from the active listening experience in the introduction to John Coltrane's *A Love Supreme: Pursuance Part III*, but that was not the case. It should be noted that the context of rhythm in more structured Western musical settings is often in relation to repeated beat patterns and meter. In the rhythmic study, there was no such context for the rhythmic events, as the sonifications placed focus only on data-derived rhythms outside of metric constraints. The Rhythm survey results may show that users expect more familiar musical sounds based on traditional roles of rhythm that would rely on repeated rhythmic patterns and hierarchical beat structures in a metric context (Bouwer et al., 2018). Evaluating sonifications in the context of rhythm appears to encompass some secondary contexts with expectations of beat pattern repetition and meter (Desain and Honing, 2003). This would require further study to validate.

The surveys show that musical characteristics in our data sonifications with D2M software contribute to engagement, but further studies are required to determine the extent to which each musical characteristic enhances listening and interpreting experiences. The most stringent analyses of the data from our three studies indicate that tones with timbre and rhythm show promise in elevating engagement, especially in the factors of *aesthetics, novelty*, and *perceived usability*. Plain sounds alone, as mostly a uniform percussion noise (from claves) with some rhythm, elevated engagement within the factor of *focused attention* (statement 23 with reference to being “absorbed” in the listening task). This preliminary finding suggests that data sonification mapping should consider the role of timbre and rhythm to enhance user experiences.

## 8. Conclusion

The aural experience of auditory display (in terms of data with sounds) can exist with or without musical characteristics. A new path for the inclusion of musical characteristics is opening up based on the influence of engagement in relation to the user experience. The design of the D2M conversion software shows promise in how musical sounds can capture the meaning of datasets and enhance the aesthetic experience, which represents a significant goal for usability and added focused time for analysis. There are also benefits to experiencing data analysis from a unique and alternative perspective (aural). Research in data-to-music has generally been tentative because of (1) the challenges of design and decision-making for musical experiences from data, (2) biases of musical tastes among users, and (3) the risks of cluttering the data message with decorative sounds. However, the potential rewards of engagement can be meaningful. Our user engagement study provides a preliminary understanding of the relevance timbre, pitch, and rhythm can have to the experience of data *via* musically informed sonifications. The characteristics of timbre and pitch, in particular, appear to have a significant impact on the aesthetics of auralization and auditory display. The studies also show that timbre and pitch, in the context of melody and chords, are influential factors in the engagement factors of *perceived usability, aesthetics*, and *novelty*, and that plain sounds can be influential for *focused attention* in the context of rhythm. Musical attributes remain inconclusive for the factors *endurability* and *felt involvement*.

This preliminary study was simplified to isolate musical traits and allow for improved observation. For real life use, we believe that a larger set of sonifications should be tested. Such sonifications should communicate information clearly and this will likely have improved results for the engagement factors *endurability* and felt involved. The observations presented in this paper set a foundation to investigate even more elaborate musical representations of data.

## Data availability statement

The datasets and audio files presented in this study can be found in online repositories. The names of the repository/repositories and accession number(s) can be found below: https://data.mendeley.com/datasets/srp4rhjkmy/2.

## Ethics statement

The studies involving human participants were reviewed and approved by Institutional Review Board: Exemption was provided by Eastern Washington University (human subjects protocol HS-5429) on December 1, 2017. The patients/participants provided their written informed consent to participate in this study.

## Author contributions

Conceptualization: JM, JHa, and RR. Methodology: JM, JHa, and PH. Software: JHa, KT, JM, and JHe. Validation: JM, KT, JHe, TK, and MZ. Formal analysis: JM, JC, and TK. Investigation: JM, KT, TK, JHe, and PH. Resources: MT and JL. Data curation: JHe and KT. Writing original draft: JM. Writing review and editing: JM, JC, and RR. Visualization and auralization: PH and JM. Supervision: RR. Project administration: MT, JL, and RR. Funding acquisition: RR and JM. All authors have read and agreed to the published version of this manuscript.

## Figures and Tables

**Figure A2:**
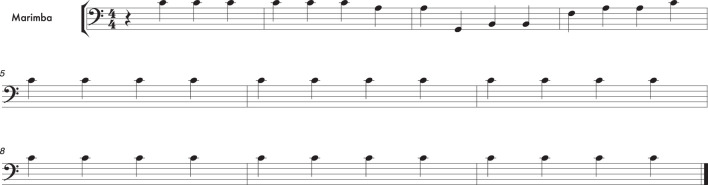
Melody generated by D2M from inverted clouds data in okta from May. Compare the melodic contour of the musical score with Figure 1 and May's clouds melodies below. The melodic contour slopes down and then back up. This was represented in the UES study as pitched pure tones, pitched timbre (marimba), and pitched timbre (marimba) with some rhythm.

## Supplementary material

**Supplementary samples of audio files from May weather datasets:**

Audio S1 May clouds data melody as pure tones.

Audio S2 May clouds data melody as pitches with timbre.

Audio S3 May clouds data melody as pitches with timbre and some rhythm.

Audio S4 May clouds data chords as pure tones.

Audio S5 May clouds data chords as pitches with timbre.

Audio S6 May clouds data chords as pitches with timbre and some rhythm.

Audio S7 May wind data as rhythms with uniform noise.

Audio S8 May wind data as rhythms with uniform pitch with timbre.

Audio S9 May wind data as rhythms with pitched timbre and noise.

## Appendix

### Data mapping and audio file standards

The oktas data, on a scale of 0–8, were mapped to a range of a musical octave and a half with a tempo of one beat per second (See sample score in **Figure A2**). A total of 40 data points in a 24-h period were displayed in 40 s. A MIDI range of 43–60 was used as a destination span, and all results adhered to a C major diatonic collection (Figure A1). A sample musical score of the May oktas data inverted for sunshine (seen in Figure 1) is provided below to show the musical range and level 1 rhythmic activity (Figure A2). The resulting MIDI files were rendered through software instruments on Logic Pro X and Ableton Live, and the resulting audio files were exported and converted to mp3 files for Web delivery with loudness normalized to negative 25 LUFS.
